# Psychological Well-Being and Positive Aspects of Caregiving: What’s the Relationship?

**DOI:** 10.1093/geroni/igaa057.1134

**Published:** 2020-12-16

**Authors:** Kelly O Malley, Sara Qualls

**Affiliations:** 1 New England GRECC, VA Boston, Boston, Massachusetts, United States; 2 University of Colorado Colorado Springs, Colorado Springs, Colorado, United States

## Abstract

Many family caregivers derive meaning and purpose from caring for a loved one. Research on the mechanisms underlying positive aspects of caregiving have explored appraisal, reciprocity, resilience, gratitude, mastery, and appreciation. This study aimed to explore how individual personality factors, using the framework of psychological well-being (i.e., autonomy, positive relationships, purpose, personal growth, self-acceptance, and environmental mastery) proposed by Ryff (1989), may influence positive aspects of caregiving. N = 452 family caregivers completed informed consent, provided demographic information, and completed study measures using an online research platform. Multiple regression equations were calculated to assess which aspects of psychological well-being predicted two positive aspects of caregiving: competence and personal gain. The model explained 22% of the variance in caregiving competence, F(6, 445) = 21.01, p < .001. Autonomy (β = .26, p < .001) and self-acceptance (β = .18, p = .02) contributed most significantly to feelings of competence. The model explained 14% of the variance in personal gain, F(6, 445) = 12.11, p < .001. Self-acceptance (β = .19, p = .02), personal growth (β = .18, p = .01) and autonomy (β = .13; p = .03) contributed most significantly feelings of personal gain. Psychological well-being, specifically feelings of autonomy and self-acceptance, are modest predictors of feelings of competence and gain, and psychological well-being appears to have an effect on positive aspects of caregiving. Interventions for family caregivers should consider ways of building autonomy, self-acceptance, and growth to increase feelings of competence and gain.

